# Evolution of re-epithelialization postcircumferential esophageal endoscopic submucosal dissection

**DOI:** 10.1016/j.vgie.2021.02.011

**Published:** 2021-03-19

**Authors:** Robert Bechara

**Affiliations:** Queens University, Kingston Health Sciences Center, Kingston, Ontario, Canada

**Keywords:** ESD, endoscopic submucosal dissection

## Abstract

Video 1 This patient with Barrett’s esophagus with multifocal high-grade dysplasia underwent complete circumferential ESD. We illustrate the evolution of re-epithelialization after circumferential esophageal ESD and the regimen used to prevent stricture formation.

This patient with Barrett’s esophagus with multifocal high-grade dysplasia underwent complete circumferential ESD. We illustrate the evolution of re-epithelialization after circumferential esophageal ESD and the regimen used to prevent stricture formation.

A 62-year-old woman with C1M5 Barrett's with multifocal high-grade dysplasia on biopsy was referred for management. Various treatment options were reviewed, including endoscopic mucosal resection, radiofrequency ablation, staged endoscopic submucosal dissection (ESD), and complete circumferential ESD. The decision was made to proceed with complete circumferential ESD.

The complete circumferential ESD was completed in 2.5 hours without any adverse events. The distal and proximal margins were normal gastric and normal squamous mucosa, respectively.

The patient was discharged 24 hours later, consuming a full fluid diet that was gradually advanced over 4 days to a normal diet. After the procedure, the patient was initiated on the following regimen:

1. Prednisone 30 mg daily for 4 weeks, then tapered by 5 mg every 2 weeks
2. Sucralfate suspension 1g 4 times daily
3. Budesonide 1 mg 4 times daily mixed with the sucralfate
4. Rabeprazole 20 mg twice daily

Figure 1 illustrates the evolution of re-epithelialization from day 0 to 32 weeks after circumferential ESD.

**Figure 1:**
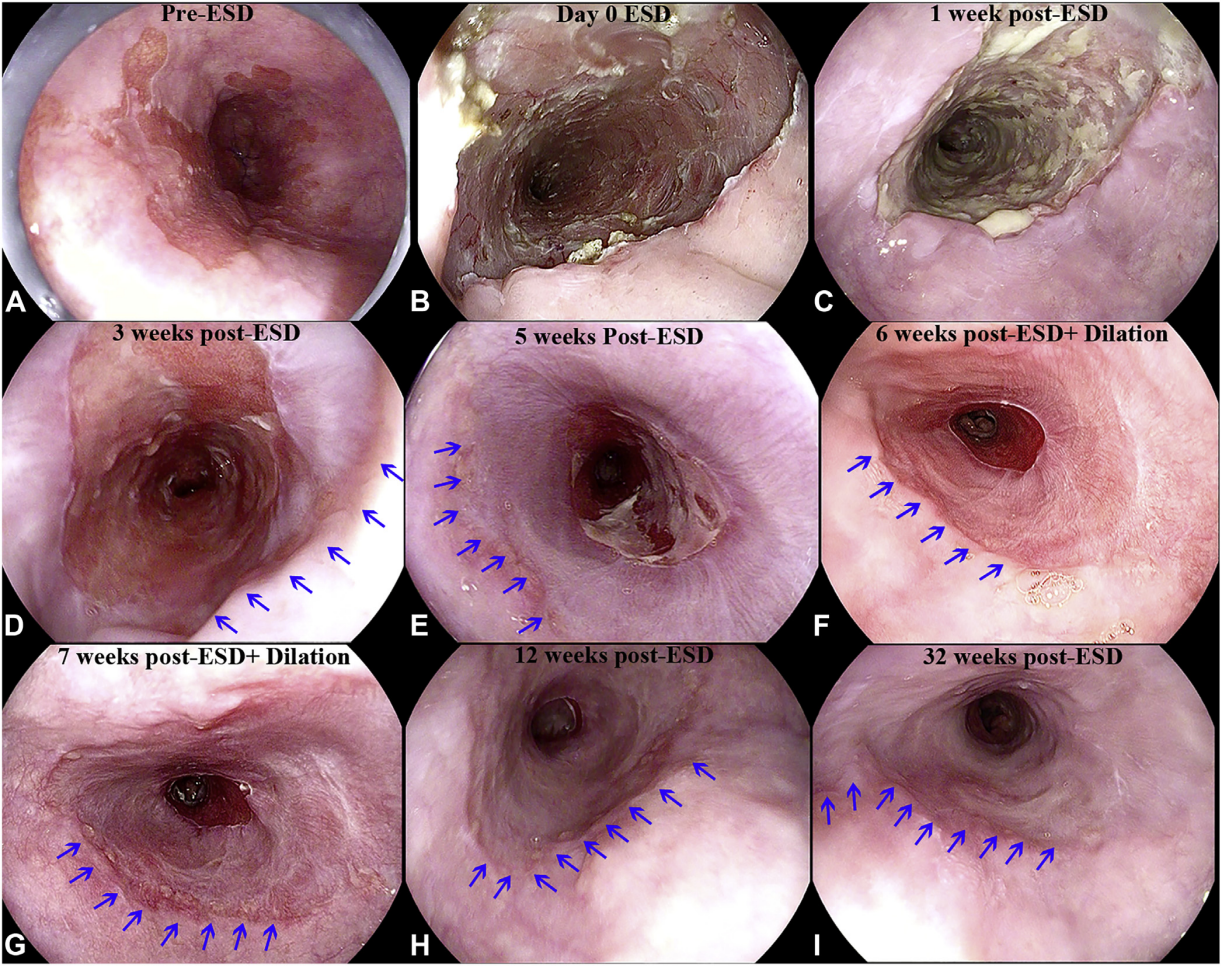
Evolution of re-epithelialization after circumferential esophageal endoscopic submucosal dissection (ESD). **A,** C1M5 Barrett’s pre-ESD. **B,** Defect immediately after ESD. **C,** One week after ESD, no significant re-epithelialization or adherent sucralfate-budesonide slurry noted. **D-I,** Progressive re-epithelization. *Blue arrows* identify the proximal squamous neosquamous border.

Complete circumferential ESD achieved R0 resection of both the dysplastic Barrett's and the entire segment of Barrett’s simultaneously. This case demonstrates the evolution of re-epithelialization after circumferential ESD. Furthermore, stricture prophylaxis was achieved with the use of prednisone, topical budesonide, sucralfate suspension, proton pump inhibitors, and 2 prophylactic balloon dilations (Video 1, available online at www.VideoGIE.org).

## Disclosure

*Dr Bechara is a consultant for Olympus and Medtronic.*

## Supplementary data

Video 1 This patient with Barrett’s esophagus with multifocal high-grade dysplasia underwent complete circumferential ESD. We illustrate the evolution of re-epithelialization after circumferential esophageal ESD and the regimen used to prevent stricture formation.

